# Parastomal Hernia: A Retrospective Nationwide Cohort Study Comparing Different Techniques with Long-Term Follow-Up

**DOI:** 10.1007/s00268-021-05990-z

**Published:** 2021-02-09

**Authors:** Elisa Mäkäräinen-Uhlbäck, Jaana Vironen, Ville Falenius, Pia Nordström, Anu Välikoski, Jyrki Kössi, Aristotelis Kechagias, Maija Kalliala, Anne Mattila, Tuomo Rantanen, Tom Scheinin, Pasi Ohtonen, Tero Rautio

**Affiliations:** 1 Department of Surgery, Medical Research Center, University of Oulu, Oulu University Hospital, 29, 90029 OYS Oulu, PL Finland; 2 Abdominal Center, Helsinki University Hospital, PL 8000, 00029 HUS Helsinki, Finland; 3 Department of Surgery, University of Turku, PL 52, 20521 Turku, Finland; 4 Department of Surgery, Tampere University Hospital, PL 2000, 3352o Tampere, Finland; 5 Department of Surgery, Päijät-Häme Central Hospital, Keskussairaalankatu 7, 15850 Lahti, Finland; 6 Department of Surgery, Kanta-Häme Central Hospital, Ahvenistontie 20, 13530 Hämeenlinna, Finland; 7 Department of Surgery, Joensuu Central Hospital, Tikkamäentie 16, 80210 Joensuu, Finland; 8 Department of Surgery, Keski-Suomi Central Hospital, Keskussairaalantie 19, 40620 Jyväskylä, Finland; 9 Department of Surgery, Kuopio University Hospital, PL 100, 70029 KYS Kuopio, Finland; 10 Division of Operative Care, Oulu University Hospital, PL 29, 90029 OYS Oulu, Finland; 11 The Research Unit of Surgery, Anesthesia and Intensive Care, University of Oulu, Oulu, Finland

## Abstract

**Background:**

Parastomal hernia repair is a complex surgical procedure with high recurrence and complication rates. This retrospective nationwide cohort study presents the results of different parastomal hernia repair techniques in Finland.

**Methods:**

All patients who underwent a primary end ostomy parastomal hernia repair in the nine participating hospitals during 2007–2017 were included in the study. The primary outcome measure was recurrence rate. Secondary outcomes were complications and re-operation rate.

**Results:**

In total, 235 primary elective parastomal hernia repairs were performed in five university hospitals and four central hospitals in Finland during 2007–2017. The major techniques used were the Sugarbaker (38.8%), keyhole (16.3%), and sandwich techniques (15.4%). In addition, a specific intra-abdominal keyhole technique with a funnel-shaped mesh was utilized in 8.3% of the techniques; other parastomal hernia repair techniques were used in 21.3% of the cases. The median follow-up time was 39.0 months (0–146, SD 35.3). The recurrence rates after the keyhole, Sugarbaker, sandwich, specific funnel-shaped mesh, and other techniques were 35.9%, 21.5%, 13.5%, 15%, and 35.3%, respectively. The overall re-operation rate was 20.4%, while complications occurred in 26.3% of patients.

**Conclusion:**

The recurrence rate after parastomal hernia repair is unacceptable in this nationwide cohort study. As PSH repair volumes are low, further multinational, randomized controlled trials and hernia registry data are needed to improve the results.

## Introduction

Parastomal hernia (PSH) is the most common complication of end colostomy with a remarkably high incidence of over 50% [1, 2]. Both the prevalence and incidence of surgical treatment are likely to increase due to better survival after rectal carcinoma treatment and the epidemic of obesity predisposing to PSH [3]. The majority of PSH cases can be treated conservatively [4–7]. However, a large number of patients with PSH have symptoms that reduce their quality of life [8–11].

PSH repair results are unsatisfactory, as the reported recurrence rate after a primary repair may be as high as 0–50%, with a high rate of surgery-related morbidity [8, 12]. In addition, no specific recommendation on the optimal repair technique exists due to lacking evidence [2, 4, 13]. The keyhole technique may lead to a recurrence rate of over 20% [14, 15]; therefore, it should be avoided, as recommended by the European Hernia Society (EHS) [2]. Suture repair and ostomy replacement have mainly been abandoned due to high recurrence rates [2, 4, 14, 15]. Meanwhile, the Sugarbaker technique, first described in 1985, is superior to the keyhole technique in both open and laparoscopic PSH repairs due to its lower recurrence rate and lack of increased risk of morbidity [2, 14–16]. Yet, according to reports and case series publications, the sandwich technique may have better outcomes compared to the keyhole and Sugarbaker techniques [12, 17–19].

A large register-based cohorts of PSH repair were previously published in Sweden [20], in Denmark using the Danish Hernia Database [21] and in the USA using the Americas Hernia Society Quality Collaborative (AHSQC) database [11]. The Swedish study reported a 27% recurrence rate and 32% complication rate after heterogeneous suture and mesh repairs, as well as relocations with and without a mesh [20]. The Danish Hernia Register study reported a 17% re-operation rate due to recurrence in three years and a 17% re-operation or morbidity rate at the 30-day follow-up [21]. The AHSQC database reported 15% surgical site occurrence at the 30-day follow-up and improved quality of life at the 2-year follow-up [12].

Thus, this nationwide cohort study aimed to report the results of different techniques used to repair end-ostomy PSH in Finland in terms of recurrence and re-operation rates during long-term follow-up, as well as complications at the 30-day and during long-term follow-up.

## Methods

This retrospective cohort study included patients who underwent elective primary PSH repair between 2007 and 2017 in all five university hospitals in Finland (Helsinki, Oulu, Turku, Tampere, and Kuopio), as well as the four central hospitals located in Lahti, Hämeenlinna, Joensuu, and Jyväskylä. The study was approved by the Audit Departments of all the participating hospitals.

### Materials

Data on a cohort of all 235 patients who had a primary PSH repair were retrieved from the hospital records using International Classification of Diseases (ICD)-10 codes combined with operation codes. The data were surveyed to identify the desired study population during the predefined period, which ranged between January 1, 2007 and December 31, 2017. The data collected in specific electronic case record forms (eCRFs) included age, body mass index (BMI), indication and date for index ostomy formation, other hernias detected during the PSH repair, technique used in the PSH surgery, mesh details, complications, length of hospital stay, re-operations, and recurrence. The primary outcome of this study was PSH recurrence during follow-up. The follow-up time was calculated from the primary operation to repair a PSH to the last date the patient was seen at the outpatient clinic. PSH recurrence is defined as a PSH detected following primary repair through either a clinical assessment by a surgeon or an imaging study. Secondary outcomes were complications at both the 30-day and during long-term follow-up and the re-operation rate during long-term follow-up.

### Statistical analysis

Summary statistics are presented as the mean and standard deviation (SD) or as the median with 25th to 75th percentiles. Between-group comparisons for continuous variables were performed using analysis of variance (ANOVA) or Welch’s test; the latter was used if the assumption of equal variances did not hold. Tukey’s test or Tamhane’s test (if the assumption of equal variances did not hold) was used as the post-test when comparing separate groups. Categorical data were analyzed using the chi-squared test or Fisher’s exact test. Kaplan–Meier survival curves were drawn, and the Tarone–Ware test was calculated for the between-group Tirone comparison to determine the recurrence of PSH. Two-tailed *p* values are presented. All analyses were performed using SPSS for Windows (version 25, IBM Corp., Armonk, NY, USA).

## Results

A nationwide cohort of 235 patients, including 68.5% (161/235) end colostomies and 31.5% (74/235) end ileostomies, who had a PSH repair were identified with a median follow-up time of 39.0 months (0–146, SD 35.3). The operations were performed by 85 surgeons at five university and four non-university hospitals. Only five surgeons operated on 10 or more PSH repairs during the 10-year study period. Each hospital’s contribution is presented in Table 1.

**Table 1:** Hospital contributions and PSH techniques used

	All (*n* = 235)	Keyhole (*n* = 39)	Sugarbaker (*n* = 91)	Sandwich (*n* = 37)	Modified keyhole (*n* = 20)	Other (*n* = 48)
Hospital 1	48 (20.4)	4 (10.3)	3 (3.3)	25 (67.6)	6 (30.0)	10 (20.8)
Hospital 2	42 (17.9)	10 (25.6)	29 (31.9)	0	1 (5.0)	2 (4.2)
Hospital 3	36 (15.3)	12 (30.8)	17 (18.7)	0	0	7 (16.6)
Hospital 4	26 (11.1)	3 (7.7)	2 (2.2)	2 (5.4)	8 (40.0)	11 (22.9)
Hospital 4	21 (8.9)	3 (7.7)	12 (13.2)	0	1 (5.0)	5 (10.4)
Hospital 5	23 (9.8)	3 (7.7)	13 (14.3)	0	3 (15.0)	4 (8.3)
Hospital 6	17 (7.2)	1 (2.6)	4 (4.4)	9 (24.3)	1 (5.0)	2 (4.2)
Hospital 8	13 (5.5)	1 (2.6)	5 (5.5)	0	0	7 (14.6)
Hospital 9	9 (3.8)	2 (5.1)	6 (6.6)	1 (2.7)	0	0
All	235 (100)	39 (16.6)	91 (38.7)	37 (15.4)	20 (8.5)	48 (20.4)

Nominal variables are reported as counts and percentages (in parentheses). The percentage indicates the portion operated using each technique

**Fig. 1:**
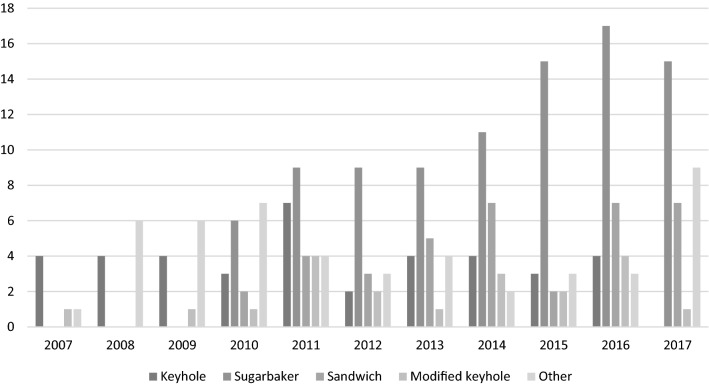
Comparison of the number of parastomal hernias repaired using different techniques: 2007–2017

The patient characteristics and operation details are presented in Table 2. There was a significant difference in the length of follow-up between the groups due to a change in the current practice during the study period (Table 2, Fig. 1). The most common technique to repair a PSH was the Sugarbaker technique, which was used in 38.5% (91/235) of all operations. The keyhole technique was utilized in 16.6% (39/235) of the PSH repairs and the sandwich technique in 15.7% (37/235). A specific funnel-shaped intra-abdominal mesh (Dynamesh IPST™, FEG Textiltechnik, Aachen, Germany) as a modification of the intra-abdominal keyhole technique, later referred to as the “modified keyhole technique,” was used in 8.5% (20/235) of the operations. In addition, there were 12 (5.1%) suture repairs, a change of stoma location in 12 (5.1%) repairs without a preventive mesh and in 10 (4.3%) repairs with a preventive mesh, six (2.6%) retrorectus mesh repairs, two (0.9%) onlay mesh repairs, and six (2.6%) non-specified mesh repairs, which are all grouped under the category “other” here and in Table 2. Due to the heterogeneity of the “other” category, the p value is calculated among the keyhole, Sugarbaker, sandwich, and modified keyhole repair techniques.

**Table 2:** Patient characteristics and operation details

	*N* total	Keyhole (*n* = 37)	Sugarbaker (*n* = 91)	Sandwich (*n* = 37)	Modified keyhole (*n* = 20)	*P* value	Other (*n* = 48)
Age (years)	235	67.1 ± 9.9	68.4 ± 10.6	70.0 ± 12.3	64.6 ± 9.9	0.30	63.5 ± 14.9
Gender	235					0.060	
Female		21 (53.8)	49 (53.8)	19 (51.4)	17 (85.0)		20 (41.7)
Male		18 (46.2)	42 (46.2)	18 (48.6)	3 (15.0)		28 (58.3)
Body mass index	174	28.3 ± 5.8	28.8 ± 5.7	28.6 ± 4.4	25.4 ± 4.5	0.17	27.1 ± 4.7
Stoma type	235					0.20	
Colostomy		25 (64.1)	71 (78.0)	23 (62.2)	15 (75.0)		27 (56.3)
Ileostomy		14 (35.9)	20 (22.0)	14 (37.8)	5 (25.0)		21 (43.8)
Indication	235					0.13	
Cancer		20 (51.3)	53 (58.2)	21 (56.8)	8 (40.0)		18 (37.5)
Inflammatory bowel disease		9 (23.1)	19 (20.9)	13 (35.1)	3 (15.0)		21 (43.8)
Diverticulosis		0	4 (4.4)	0	3 (15.0)		2 (4.2)
Anal incontinence		4 (10.3)	7 (7.5)	1 (2.7)	3 (15.0)		1 (2.1)
Other		6 (15.4)	8 (8.6)	2 (5.4)	3 (15.0)		6 (12.5)
Follow-up (months)	235	53.3 ± 37.1	33.2 ± 28.7	49.2 ± 29.0	49.5 ± 37.8	0.002	56.2 ± 42.5
Time (months) from primary operation to hernia repair	216	80.7 ± 88.4	79.2 ± 83.3	144.1 ± 175.8	88.3 ± 122.8	0.34	95.7 ± 118.3
Time (months) from hernia repair to recurrence	59	29.2 ± 36.1	22.5 ± 17.6	35.0 ± 26.0	12.0 ± 11.0	0.57	22.5 ± 28.9
Operation	235					< 0.001	
Laparoscopic		11 (28.2)	68 (74.7)	31 (83.8)	6 (30.0)		7 (14.6)
Open		24 (61.5)	14 (15.4)	4 (10.8)	12 (60.0)		40 (83.3)
Hybrid		4 (10.3)	9 (9.9)	2 (5.4)	2 (10.0)		1 (2.1)
Operation duration (min)	147	123.0 ± 58.9	132.4 ± 70.6	126.4 ± 67.7	121.2 ± 52.0	> 0.90	110.8 ± 56.2
Blood loss (mL)	163	141.4 ± 279.4	64.2 ± 105.5	38.8 ± 41.7	82.2 ± 51.6	0.006	77.6 ± 111.1
Mesh material	204					< 0.001	
Polypropylene		18 (48.6)	11 (12.1)	5 (13.5)	0		10 (20.8)
Polyester		4 (10.8)	67 (73.6)	3 (8.1)	0		3 (6.3)
Polyvinylidene fluoride		13 (35.1)	9 (9.9)	26 (70.3)	20 (100.0)		10 (20.8)
Biologic		1 (2.7)	0	0	0		1 (2.8)
Mesh not known		3 (8.1)	4 (4.4)	3 (8.1)	0		0
Size of the mesh (cm^2^)	179	249.9 ± 98.9	272.0 ± 100.7	371.3 ± 181.4	286.3 ± 191.2	0.016	272.1 ± 212.8
Other ventral hernia	235	5 (12.8)	13 (14.3)	7 (18.9)	2 (10.0)	0.84	6 (12.5)
Length of stay in hospital	226	7.5 ± 5.2	6.9 ± 7.1	6.3 ± 5.1	6.5 ± 3.8	0.85	8.2 ± 5.6

Nominal variables are reported as counts and percentages (in parentheses); continuous variables are reported as mean and standard deviation. *P* value is calculated among the keyhole, Sugarbaker, sandwich, and modified keyhole techniques

The overall rate of laparoscopic operations was 52.3% (123/235). However, mini-invasive laparoscopic or robotic surgery was used in 74.7% (68/91) of the PSH repairs using the Sugarbaker technique and 83.8% (31/37) of the repairs using the sandwich technique (Table 2).

**Fig. 2:**
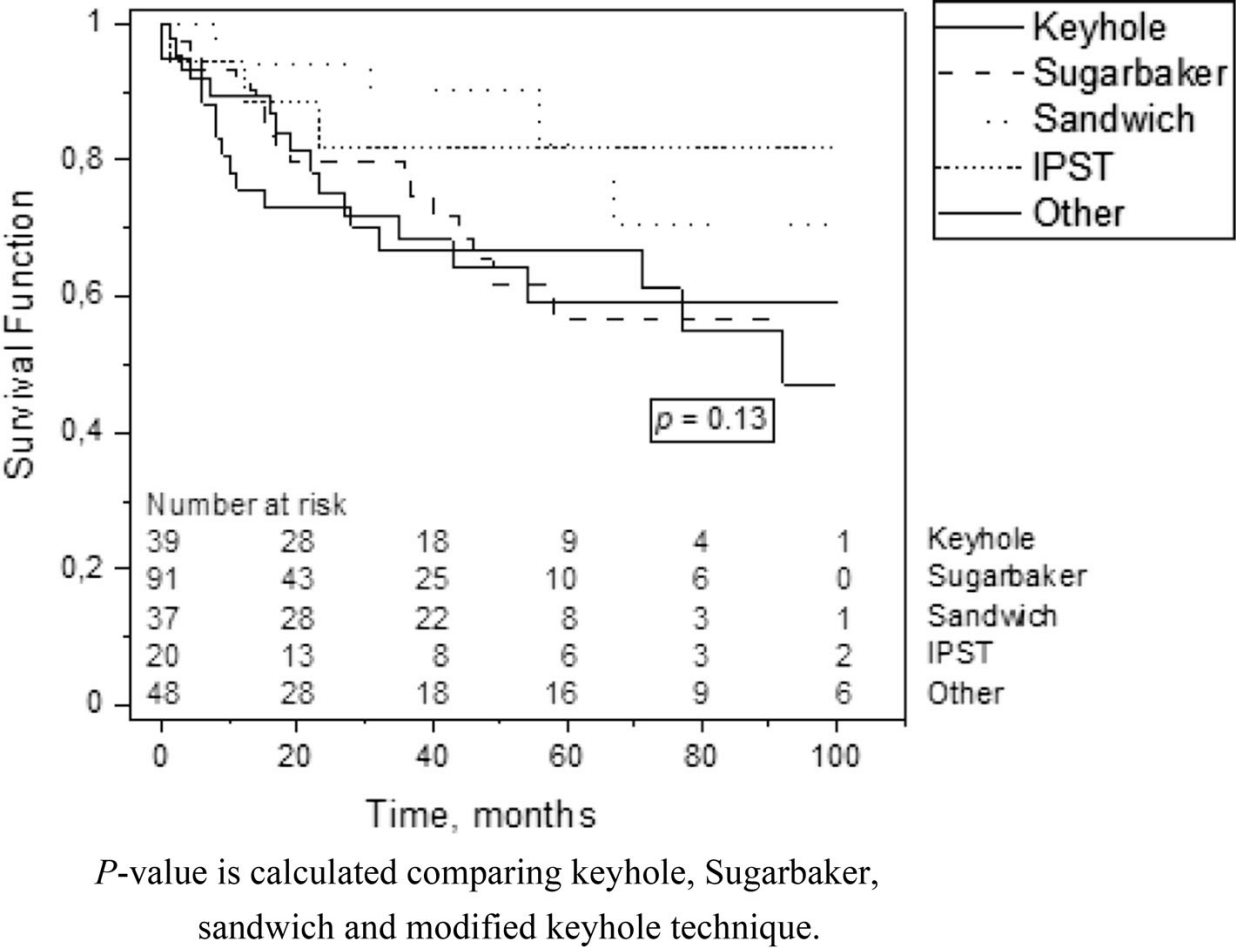
Parastomal hernia recurrence after primary repair. *P* value is calculated comparing keyhole, Sugarbaker, sandwich and modified keyhole technique

The overall recurrence rate was 24.7%. The recurrence rate was 35.9% for the keyhole technique, 21.5% for the Sugarbaker technique, 13.5% for the sandwich technique, and 15.0% for the modified keyhole technique (p = 0.11); meanwhile, it was 35.3% (16/48) for the techniques in the “other” category (Table 3). The median time from primary PSH repair to recurrence was 24.6 months (0–142, SD 26.5), with no difference between the different repair techniques (*p* = 0.573). The Kaplan–Meier curve demonstrates the timeline of recurrence after the primary PSH operation (Fig. 2; p = 0.158).

**Table 3:** Parastomal hernia repair results

	Keyhole (*n* = 39)	Sugarbaker (*n* = 91)	Sandwich (*n* = 37)	Modified keyhole (*n* = 20)	*P* value	Other (*n* = 48)
Recurrence	14 (35.9)	20 (22.0)	5 (13.5)	3 (15.0)	0.11	16 (33.3)
Re-operation	9 (23.1)	17 (18.7)	3 (8.1)	3 (15.0)	0.03	16 (33.3)
Parastomal hernia recurrence	3 (33.3)	11 (68.8)	0	2 (66.7)		8 (50.0)
Prolapse	1 (11.1)	0	0	0		4 (25.0)
Fistula	1 (11.1)	0	00	0		1 (6.3)
Infection, mesh removed	0	0	2 (66.7)	0		0
Stricture	0	1 (5.9)	0	1 (33.3)		0
Seroma	0	0	1 (33.3)	0		0
Unknown	5 (55.6)	5 (29.4)	0	0		3 (18.8)
Complications					0.53	
Complications 30 days						
Surgical site infection (SSI)	4 (10.3)	11 (12.1)	5 (13.5)	0		2 (4.2)
Other infection	3 (7.7)	6 (6.6)	2 (5.4)	0		2 (4.2)
Bleeding complication	2 (5.1)	5 (5.5)	0	0		3 (6.3)
Cardiovascular complication	1 (2.6)	2 (2.2)	0	0		0
Thromboembolic complication	1 (2.6)	3 (3.3)	0	0		0
Complications during follow-up						
Small bowel obstruction	2 (5.1)	6 (6.6)	3 (8.1)	3 (15.0)		6 (12.5)
Fistula	3 (7.7)	3 (7.7)	0	0		1 (2.1)

Nominal variables are reported as counts and percentages (in parentheses). *P* value is calculated comparing the keyhole, Sugarbaker, sandwich, and modified keyhole techniques due to the heterogeneity of the “other” category

The overall complication rate was 26.4%. The complications are presented in detail in Table 3. During the follow-up period, a re-operation was performed in 23.1%, 18.7%, 8.1%, 15.0% (p = 0.03), and 39.2% of patients after the keyhole, Sugarbaker, sandwich, modified keyhole, and “other” techniques, respectively. The total re-operation rate was 20.4%. The most common indication for re-operation was PSH recurrence (Table 3).

The laparoscopic and open techniques were compared using a subgroup analysis in Table 4. The keyhole and Sugarbaker by laparoscope techniques seemed to have an increased trend of recurrence, re-operations, and complications (Table 4). On the contrary, all the fistulas occurred after open repair.

**Table 4:** Results of the parastomal hernia repair – laparoscopic versus open surgery

	Keyhole (*n* = 39)	Sugarbaker (*n* = 91)	Sandwich (*n* = 37)	Modified keyhole (*n* = 20)	*P* value	Other (*n* = 48)
Laparoscopic	11 (28.2)	68 (74.7)	31 (83.8)	6 (30.0)		7 (14.6)
Open	24 (61.5)	14 (15.4)	4 (10.8)	12 (60.0)		40 (83.3)
Recurrence					0.659	
Laparoscopic	8 (72.7)	17 (25.0)	4 (12.9)	1 (16.7)		2 (28.6)
Open	5 (20.8)	1 (7.1)	0	2 (16.7)		14 (35.0)
Re-operation					0.072	
Laparoscopic	5 (45.5)	14 (20.6)	3 (9.7)	1 (16.7)		2 (28.6)
Open	3 (12.5)	1 (7.1)	0	2 (16.7)		14 (35.0)
Complications 30 days					0.897	
Laparoscopic						
Surgical site infection (SSI)	3 (27.3)	18 (26.5)	7 (22.6)	1 (16.7)		0
Other infection	0	6 (8.8)	2 (6.5)	0		0
Bleeding	1 (9.1)	4 (5.9)	0	0		0
Cardiovascular complication	0	1 (1.5)	0	0		0
Thromboembolic complication	0	3 (4.4)	0	0		0
Open						
Surgical site infection (SSI)	3 (12.5)	3 (21.4)	1 (25.0)	0		2 (5.0)
Other infection	3 (12.5)	2 (14.3)	0	0		1 (2.5)
Bleeding	1 (4.2)	0	0	0		3 (7.5)
Cardiovascular complication	0	1 (7.1)	0	0		0
Thromboembolic complication	1 (4.2)	0	0	0		0
Complications during follow-up						
Laparoscopic						
Small bowel obstruction	1 (9.1)	3 (4.4)	2 (6.5)	1 (16.7)		0
Fistula	0	0	0	0		0
Open						
Small bowel obstruction	0	3 (21.4)	0	2 (16.7)		6 (15.0)
Fistula	3 (12.5)	2 (14.3)	0	0		1 (2.5)

Nominal variables are reported as counts and percentages (in parentheses). Percentages are calculated as portions of a given technique. *P* value is calculated comparing the keyhole, Sugarbaker, sandwich, and modified keyhole techniques due to the heterogeneity of the “other” category

## Discussion

The results of this nationwide cohort study show the grim reality of the current state of PSH repair in Finland, with a 24.7% overall recurrence rate, a 20.4% re-operation rate, and a 26.4% complication rate (Table 3). The current study revealed that both the institutional volumes and volumes per surgeon in PSH repair are low.

This study has several limitations due to the retrospective nature and small number of patients operated on using each technique. Therefore, we cannot draw any strong conclusions about the superiority or inferiority of any technique. Because the retrospective data were collected from patient registries, contributors behind the decision to choose the repair method could not be reliably assessed, nor was there an indication for PSH repair. As the EHS parastomal hernia classification is not used routinely in clinical practice, the classification was not assessed in relation to outcomes either. In addition, patient-related risk factors and comorbidities predisposing patients to both recurrence and complications remain elusive, as do patient-reported outcomes. Furthermore, the explicit indication for re-operation could not be confirmed for 10 patients. The severity of complications, i.e., Clavien-Dindo classification of complications, remains unelaborated. The strength of the study is the collection of multicenter nationwide data with a sufficient follow-up interval, likely to reflect the real-life results of PSH surgery in Finland.

Patients operated on by the laparoscopic keyhole and Sugarbaker techniques showed a trend of increased recurrence and re-operation rates compared to the open technique. Such a trend was not observed in patients who had a PSH repaired by the modified keyhole technique. A specific funnel-shaped intra-abdominal mesh (Dynamesh IPST™) as a modification of the keyhole technique has been efficient in previously published case series with recurrence rates of 0–12.5% [22, 23]. This is in line with our results, where this repair technique had a low recurrence (15.0%) and complication rate (15.0%). More studies are needed to evaluate further both the modified keyhole technique and the complexity of mini-invasive PSH repair.

The recurrence rate after keyhole repair was 35%, which is an increase over the rate previously reported [14, 15]. The PSH repair results following both the Sugarbaker and sandwich techniques were previously reported to be significantly better than that of the keyhole technique [2, 14, 15, 17, 18]. In our study, the recurrence rate after Sugarbaker repair was 21.5%, in contrast with the 10.2–15.0% rate reported in previous meta-analyses [14, 15]. Similarly, in our cohort, the recurrence rate after using the sandwich repair technique was 13.5%, in contrast to 2.0–4.8% in previous reports [12, 17–19]. The results may reflect the reality outside highly specialized abdominal wall centers.

PSH repair is prone to complications. The overall complication rate of this cohort is 26.3%, which is in line with previous studies [14, 15]. As the number of patients operated on using each technique is highly limited, no firm conclusions can be given concerning the risks of any technique. A small bowel obstruction seems a common long-term complication after PSH repair (Tables 3, 4). However, a small bowel obstruction rarely led to re-operation (Table 3).

One-fifth of all patients underwent re-operation, mainly due to recurrence or other stoma-related long-term complications. An increased trend of re-operation after laparoscopic repair compared to open repair was noted, without statistical significance (Table 4). The re-operation rate is in line with that previously reported [21]. The incidence of concomitant incisional hernia (Table 2) was exceptionally low compared to that previously reported [24]. The reasons behind the low incidence remain speculative, but may be at least partially explained by the high rate of mini-invasive operative strategies in Finland.

Because the number of institutional PSH repairs is low, international register-based studies and multicenter trials are needed to gather reliable data to guide PSH treatment and obtain enough evidence to establish international PSH treatment guidelines. PSH prevention is recommended [2, 4] but still inadequately and rarely utilized [25, 26]. Furthermore, the effectiveness of PSH prevention has been questioned [27, 28]. Therefore, novel, safe, and efficient techniques to repair PSH are demanded [24, 29], but improvements to the currently widely utilized Sugarbaker, sandwich, and modified keyhole techniques are also needed. Furthermore, more knowledge of the patient-reported outcomes of PSH repair is required to guide decisions.

## Conclusion

The rates of PSH recurrence, complications, and re-operations are unacceptably high. As PSH repair volumes are low, further multinational, randomized controlled trials and hernia registry data are needed to improve the results of surgical treatment for this condition.

## Data Availability

The datasets generated and/or analyzed during the current study are not publicly available due to Finnish laws on privacy protection; however, they are available from the corresponding author upon reasonable request.
